# Emotional predictors of bowel screening: the avoidance-promoting role of fear, embarrassment, and disgust

**DOI:** 10.1186/s12885-018-4423-5

**Published:** 2018-05-03

**Authors:** Lisa M. Reynolds, Ian P. Bissett, Nathan S. Consedine

**Affiliations:** 1 Department of Psychological Medicine, The University of Auckland, Private bag 92019, Victoria Street West, Auckland, 1142 New Zealand; 2 Department of Surgery, The University of Auckland, Private Bag 92019, Victoria Street West, Auckland, 1142 New Zealand

**Keywords:** Screening, Colorectal cancer, Emotion, Disgust, Fear, Embarrassment, Avoidance

## Abstract

**Background:**

Despite considerable efforts to address practical barriers, colorectal cancer screening numbers are often low. People do not always act rationally, and investigating emotions may offer insight into the avoidance of screening. The current work assessed whether fear, embarrassment, and disgust predicted colorectal cancer screening avoidance.

**Methods:**

A community sample (*N* = 306) aged 45+ completed a questionnaire assessing colorectal cancer screening history and the extent that perceptions of cancer risk, colorectal cancer knowledge, doctor discussions, and a specifically developed scale, the Emotional Barriers to Bowel Screening (EBBS), were associated with previous screening behaviours and anticipated bowel health decision-making.

**Results:**

Step-wise logistic regression models revealed that a decision to delay seeking healthcare in the hypothetical presence of bowel symptoms was less likely in people who had discussed risk with their doctor, whereas greater colorectal cancer knowledge and greater fear of a negative outcome predicted greater likelihood of delay. Having previously provided a faecal sample was predicted by discussions about risk with a doctor, older age, and greater embarrassment, whereas perceptions of lower risk predicted a lower likelihood. Likewise, greater insertion disgust predicted a lower likelihood of having had an invasive bowel screening test in the previous 5 years.

**Conclusions:**

Alongside medical and demographic factors, fear, embarrassment and disgust are worthy of consideration in colorectal cancer screening. Understanding how specific emotions impact screening decisions and behaviour is an important direction for future work and has potential to inform screening development and communications in bowel health.

## Background

Colorectal cancer (CRC) is the third most common cancer worldwide and the fourth leading cause of cancer-related deaths [1]. CRC screening has been shown to reduce incidence and mortality through the early identification of cancer and high risk polyps [2]. In recent years, many organised population-based screening programmes have been implemented or are being progressively rolled out across regions [3]. Screening guidelines vary but generally concur insofar as they suggest that organised programmes should utilise a combination of annual/bi-annual testing for blood in the stool (e.g., FOBT) and less frequent bowel scope screening (e.g., colonoscopy) from the age of approximately 50 in average risk persons [4, 5]. Considerable efforts have been directed towards facilitating uptake of such screening. A systematic review of interventions aimed at increasing access to screening suggest a global focus on reducing structural barriers to screening tests including driving mobile units to worksites and communities, reducing administrative demands, and offering screenings at subsidised rates or for no charge [6]. However, despite established benefits, even when screenings are free and accessible, many people continue to avoid them [7]. Participation in CRC screening programmes varies widely and a recent review of international uptake for organised screening programmes reported that participation ranged from 7 to 68% [8]. A parallel line of study has evaluated how emotions-based research may increase understanding of why people reject medical investigations they ought to attend [9]. Below, we continue in this endeavour, presenting a report investigating possible emotional (fear, embarrassment, and disgust) barriers to colorectal cancer (CRC) screening where, despite the benefits of early detection [10], screening rates are low.

There are strong reasons to suspect that the avoidance-promoting emotions of fear, embarrassment, and the less-studied disgust may act as barriers to CRC screening. In the evolutionary view, fear, embarrassment, and disgust are all emotions that specifically evolved to promote avoidance. Functionally, fear reduces danger by urging us to flee [9], embarrassment reduces the threat of exclusion by forestalling social norm violation [11], and disgust promotes health by motivating the avoidance of potential contaminants [12]. Prima facie, most CRC screenings and medical investigations involve exposure to the elicitors of all three of these feelings. Colonoscopies, for example, involve inserting apparatus into the anus and may involve pain and the threat of a cancer diagnosis (fear), the display of private body parts (embarrassment), and exposure to potential contaminants like faeces (disgust).

Fear and embarrassment have received some empirical attention in prior CRC work; fear of a cancer diagnosis [13, 14] and embarrassment from intimacy [15], especially where the physician is of a different gender [15], predict lower CRC screening. While disgust has been mostly overlooked, there are a few recent studies implying disgust-generated avoidance in CRC screening [12, 16, 17]. However, while studies of fear, embarrassment and disgust are developing, individual studies have typically focussed on single avoidance-promoting emotions in isolation without considering multiple emotions concurrently. This is problematic as the prospect of an anal investigation might *simultaneously* make a person embarrassed, fearful, and disgusted; given that the emotions reliably co-vary, measuring only one emotion may obscure the “active” emotional ingredient(s). Thus, the primary aim of this study was to investigate which of these three emotions—all of which evolved to promote avoidance—predicted CRC screening behaviours and decisions and whether one emotion might be a more salient predictor in this context.

A secondary objective of this work was to examine whether these three emotions arise with respect to specific types of CRC stimuli or tests. All emotions arise in response to specific stimuli [15, 18], with screening unlikely to be an exception. Colorectal cancer investigations typically involve differential exposure to the known elicitors of fear (e.g., of a possible cancer diagnosis), embarrassment (e.g., bodily exposure) and disgust (e.g., insertions, exposure to contaminants), suggesting different emotions may be relevant to different aspects of screening. Identifying specific avoidance-promoting emotions in colorectal cancer screening contexts may thus help identify the specific elements of the various screens that people are actually avoiding.

The avoidance-promoting emotions of fear, embarrassment, and disgust have intuitive links to CRC screening. However, disgust remains understudied and these three emotions have typically been examined in isolation from one another. The current report assessed the extent to which fear, embarrassment, and disgust predicted avoidance, over and above known confounds, in contexts related to CRC screening.

## Methods

People aged 45+ years and fluent in English were invited to take part in a study on “Emotions and Human Behaviour”. Convenience sampling was used to recruit participants using a combination of flyers posted on university and hospital notice boards, workplace emails, a letterbox drop, and an advertisement in a community newspaper. Participants were offered a $10 petrol voucher as remuneration. People who were non-English speaking and aged less than 45 years were excluded from the study.

### Measures

#### Demographic and medical factors

Demographic data together with information regarding whether participants had a regular doctor, and whether a doctor had ever discussed their risk or family history of bowel cancer were gathered.

#### CRC screening knowledge

A measure developed to assess knowledge about the United Kingdom CRC screening programme [19] was adapted for local relevance. Participants rate 12 statements related to bowel symptoms and screening as true or false indicators. Correct answers are scored as 1 and items summed to give a total.

#### Perceptions of CRC risk

Participants were asked whether they believed their risk of bowel cancer was ‘lower’, ‘about the same’, or ‘higher’ than other men/women their own age as originally used by Weinstein [20]. For the purposes of multivariate analyses, dummy codes were created for lower than average risk (yes =1, no = 0) and higher than average risk (yes =1, no = 0).

#### Disgust propensity and sensitivity-revised scale [DPSS-R; [21]]

The DPSS-R is a 12-item scale measuring propensity (how easily one is disgusted) and sensitivity (how unpleasant someone finds the disgust experience). Previous work has shown the DPSS-R to be valid and reliable and a predictor of health-related avoidance [21, 22]. Internal reliabilities in this sample were good (sensitivity α = .78; propensity α =.80).

#### Disgust sensitivity-revised scale [DS-R; [23]]

Whereas the DPSS-R assesses propensity and sensitivity of disgust, the DS-R discriminates between different types of disgust elicitors – namely animal-reminder, contamination, and core disgust. The 27-item measure asks participants to rate from 0 to 4 how disgusting they find a variety of experiences and calculates mean scores for three subscales (animal-reminder, contamination, and core); higher scores indicate greater disgust sensitivity for each of these sources. The DS-R has good internal consistency and validity [24–26], and predicts disgust-generated avoidance in experimental work investigating avoidance in colorectal cancer contexts [25, 27, 28]. Internal consistency was adequate in the current study; core α =.79, animal-reminder α =.73, contamination α =.66.

#### Differential emotions scale [DES; [29]]

The DES is a 30-item scale containing three items for each of 10 trait emotions. Using a 1 to 5 scale, participants rate the frequency with which they experience each emotion in their daily life. Mean scores for each subscale provide a score for individual emotions, with higher scores indicating more frequent experiences of that emotion. The disgust, fear, and shame subscales of the DES have been associated with CRC embarrassment [15]. Internal reliability for these subscales were adequate (shame α = .76, fear α = .88, disgust α = .70).

#### Emotional barriers to bowel screening (EBBS)

Given the absence of a measure to specifically assess *multiple* emotional barriers to CRC screening, a scale was developed for the study. Development of the EBBS was based on theory suggesting that emotions of fear, embarrassment, and disgust were of greatest relevance [15, 30]. Twenty items, each assessing one specific emotional barrier to CRC screening were developed by an experienced team of clinicians, psychologists, and researchers. Participants rate between 1 and 5 the extent to which they experience each emotion: ‘faecal’ disgust (generated by faecal exposure); insertion disgust (elicited by insertion of objects into the anus); embarrassment (related to the social context of screening); and fear of a negative outcome (worries about the potential for a cancer diagnosis). Earlier presentation of preliminary findings using the EBBS led to it being used in another study which suggested the EBBS may be unidimensional [31]. However, confirmatory factor analysis in the current sample using Maximum Likelihood extraction and Direct Oblimin rotation revealed four components with eigenvalues over .70. These components were retained as recommended by Jolliffe [32]. Five items were culled because they co-loaded on to more than one factor and/or had loadings which were less than 0.5. The 15 remaining items all loaded in a coherent way on to their respective factors (see Table 1) and multicollinearity was within acceptable levels. The overall model fit was significant χ^2^ (20) = 399.48, *p =* .000. Mean scores were calculated for each subscale. Scales had good reliability; faecal disgust (α = .90), insertion disgust (α = .94), fear of outcome (α = .84), and embarrassment (α = .89).

**Table 1:** Factor loadings based on maximum likelihood extraction with direct oblimin rotation for the EBBS

		Fear of outcome	Embarrassment	Insertion disgust	Faecal disgust
1	Collecting a sample of my own faeces/poo on a swab stick is really nasty				.658
2	The idea of posting a sample of my faeces to my doctor or a laboratory is embarrassing				
3	The idea of putting anything, even diagnostic equipment into my anus is disgusting			−.630	
4	I am afraid that giving a stool sample might lead to discovering I have cancer	.831			
5	I would be so concerned about being diagnosed with bowel cancer that I would avoid testing	.641			
6	The idea of having to gather a sample of my own faeces makes me feel sick				.835
7	Having anything guided up my bottom and into my bowel is disgusting			−.886	
8	Holding a container of my own faeces (poo) is disgusting				.578
9	I would worry that stool testing would find something wrong with me	.948			
10	Giving a stool/faecal sample for testing is really embarrassing		.507		
11	The idea of having a short, lighted tube inserted into my anus is gross			−.960	
12	Imagining that other people know I am collecting stool samples for testing would be embarrassing		.780		
13	Collecting my own faeces/poo is so disgusting I think I would gag				.582
14	I feel uncomfortable when a doctor or nurse describes bowel screening				
15	I feel anxious when I think about conducting a stool test				
16	Imagining the feeling of having a screening instrument inserted into my rectum makes me feel ill			−.822	
17	I would feel humiliated if someone found a faecal collection kit I had been given		.715		
18	The possibility that collecting faeces for a bowel test might be a bit messy is revolting				
19	I am afraid that I might hurt myself somehow during stool collection				
20	Having to insert preparatory medications into my bottom before a bowel cancer test is disgusting			−.566	

*Note*. Loadings of less than .5 are suppressed

#### Hypothetical medical help seeking

To investigate the possible role of discrete emotional barriers to bowel screening, participants were asked to imagine a scenario where they had been feeling unwell with symptoms for 5 days, with diarrhoea and a dull stomach pain and possibly blood in their stool. The scenario required a decision on making an appointment, knowing that collection of a faecal sample (a disgust elicitor) would be required prior to attendance. Participants were given the option to ‘call and make an appointment today’ (coded 0) or ‘wait another few days’ (coded 1).

#### Bowel screening history

Participants indicated whether they had *ever* provided a faecal sample for testing. They also indicated whether they had an invasive bowel screening investigation (i.e., colonoscopy, sigmoidoscopy or CT colonography) in the past 5 years. All responses were coded as no = 0 and yes = 1. The sum of the number of reported invasive bowel screening tests was used to create a composite score. As might be expected, data were skewed, hence participants were categorised as having had an invasive bowel screening test or not in the past 5 years.

### Analyses

Statistical analyses began by assessing the concurrent and discriminant validity of the EBBS subscales. Next, relationships among the measures of emotion using Pearson’s correlations were assessed using Bonferroni adjusted alpha levels of .001 per test (.05/38). Relationships between emotion measures and screening outcomes were conducted using Spearman’s rho (outcome variables were all non-normally distributed). Next, to investigate whether fear of outcome, embarrassment, or disgust predicted screening, a series of step-wise logistic regression models were run assessing whether participants a) chose to delay or not in the medical help-seeking scenario, b) had ever provided a faecal sample, and c) had an invasive bowel screening test in the past 5 years. Given the importance of age to the frequency of medical investigation and the known gender differences in bowel screening uptake [15], age and sex were entered as potential confounds at Step 1, as were other variables previously shown to predict screening including subjective risk and whether participants had discussed bowel cancer risk with a doctor [33]. At Step 2, the four EBBS subscales were entered.

## Results

The questionnaire was completed by 306 people (Table 2) with the majority of data (84%; *n* = 257) collected over twelve months (Nov 2011 to Nov 2012). A further 48 participants completed their questionnaires sixteen months later over 2 days in March 2014 following a newspaper interview with one of the study researchers.

**Table 2:** Study measures

Measure	
Age: Mean (SD)	57.07 (8.40)
Gender:	
Male	72 (23.6%)
Female	233 (76.4%)
Marital status:	
Single	35 (11.5%)
Married/cohabiting	201 (65.9%)
Separated/divorced	52 (17.0%)
Widowed	16 (5.2%)
Ethnicity:	
NZ European	244 (80.0%)
NZ Maori	15 (4.9%)
Pacific	10 (3.3%)
Asian	12 (3.9%)
Other	24 (7.9%)
Completed an invasive bowel screening test in last five years:	
Colonoscopy	28 (9.2%)
Sigmoidoscopy	67 (22.0%)
CT colonography	24 (7.9%)
Ever provided a faecal sample for testing	130 (42.6%)

Participants were offered the opportunity to complete the questionnaire either online or in written form; the majority chose the online format (88.2%, *n* = 269) which was accessed through either an electronic link (i.e., emails, online newspaper article) or the URL specified in print format (i.e., flyers, newspaper advertisement). Of the 298 people who commenced the online survey, 90.3% (*n* = 269) completed the questionnaire. One person was excluded from analyses because of a prior diagnosis of CRC. Ages ranged between 45 and 88 years (median = 55 years) with the majority being female (77%).

As is common, measures of emotion were correlated (Table 3). However, greater discrimination was revealed when we examined associations between emotions and the CRC screening variables. Consistent with theory suggesting that emotions related to specific screening elicitors would be better predictors than general dispositions [15, 18], EBBS subscales were more consistently and more strongly associated with self-reported bowel health outcomes than the more general measures of emotion (i.e., the DS-R, DPSS-R, and the DES subscales; see Table 4). Given the relative strength of these links, multivariate analyses proceeded with the EBBS subscales alone.

**Table 3:** Pearson correlations, means and standard deviations of emotion measures

Measure	Mean	SD	Faecal disgust	Insertion disgust	Fear of outcome	Embarrassment
EBBS subscales:						
Faecal disgust	1.98	0.98				
Insertion disgust	2.09	1.05	0.80*			
Fear of outcome	1.80	0.94	0.51*	0.51*		
Embarrassment	1.95	1.02	0.78*	0.74*	0.51*	
DS-R subscales:						
Core	2.05	0.67	0.44*	0.40*	0.23*	0.42*
Animal-reminder	1.62	0.78	0.43*	0.38*	0.29*	0.36*
Contamination	1.40	0.80	0.39*	0.37*	0.27*	0.33*
DPSS-R subscales:						
Propensity	2.58	0.52	0.46*	0.42*	0.32*	0.39*
Sensitivity	1.97	0.62	0.39*	0.38*	0.32*	0.40*
DES subscales:						
Fear	5.87	2.38	0.24*	0.26*	0.21*	0.29*
Disgust	5.56	1.93	0.36*	0.42*	0.34*	0.32*
Shame	6.11	2.18	0.31*	0.31*	0.29*	0.29*

*p < .001

**Table 4:** Spearman’s rho correlations between emotion measures and bowel screening behaviours

Measure	Decision to delay seeking medical help	Ever provided a faecal sample	Invasive bowel screening test in last 5 years
EBBS subscales:			
Faecal disgust	0.31**	−0.13^*^	−0.12*
Insertion disgust	0.28**	−0.18**	−0.24**
Fear of outcome	0.31**	− 0.09	− 0.13^*^
Embarrassment	0.32**	− 0.04	− 0.16**
DS-R subscales:			
Core	0.07	− 0.02	− 0.05
Animal-reminder	0.08	−0.11^+^	− 0.07
Contamination	0.09	−0.03	0.02
DPSS-R subscales:			
Propensity	0.10^+^	0.06	−0.07
Sensitivity	0.14*	−0.02	−0.07
DES subscales:			
Fear	0.15*	0.00	0.04
Disgust	0.14*	−0.16*	−0.04
Shame	0.19**	−0.05	−0.04

**p < .01, *p < .05, ^+^*p* < .10

In the first model, the predictors of medical help-seeking in the hypothetical presence of bowel symptoms were assessed. Whilst the model was not significant at Step 1, χ^2^ (2, *N* = 303) = 10.71, *p =* .098, it was significant with the addition of the EBBS subscales at Step 2, χ^2^ = 57.88, *p = .*000 (Table 5) and explained 24.3% of the variance in delays to help seeking (Nagelkerke *R*^*2*^). Having ever had a discussion with a doctor about colorectal cancer risk predicted lower odds of delaying medical help-seeking (Wald = 4.45, *p* = .035), whereas greater CRC screening knowledge predicted a greater likelihood of deciding to ‘wait another few days’ before making an appointment (Wald = 3.98, *p* = .046). Greater fear of a negative outcome predicted greater likelihood of delay (Wald = 6.29, *p* = .012), and screening embarrassment showed a trend in the same direction (Wald = 3.28, *p* = .070).

**Table 5:** Step-wise logistic regression: Final (Step 2) models showing the multivariate predictors of bowel screening behaviours

	Decision to delay seeking medical help		Ever provided a faecal sample		Invasive bowel screening test in past 5 years	
Variable	Odds Ratio (95% CI)	*p* value	Odds Ratio (95% CI)	*p* value	Odds Ratio (95% CI)	*p* value
Sex^1^	1.22 (0.62–2.39)	.560	1.80 (0.98–3.31)	.059	1.84 (0.85–3.97)	.123
Age	1.00 (0.97–1.04)	.843	1.05 (1.02–1.08)	.002	1.06 (1.02–1.10)	.002
Perceived low colorectal cancer risk^2^	1.31 (0.68–2.54)	.418	0.51 (0.27–0.96)	.038	0.52 (0.22–1.22)	.133
Perceived high colorectal cancer risk^3^	1.06 (0.43–2.62)	.906	0.51 (0.23–1.16)	.107	1.76 (0.71–4.36)	.225
Discussed with doctor^4^	0.47 (0.24–0.95)	.035	2.16 (1.21–3.85)	.009	6.79 (3.57–12.94)	.000
Colorectal cancer screening knowledge	1.19 (1.00–1.42)	.046	0.99 (0.85–1.15)	.868	1.14 (0.93–1.38)	.206
EBBS fear of outcome	1.53 (1.10–2.13)	.012	0.89 (0.64–1.24)	.484	0.92 (0.60–1.42)	.718
EBBS embarrassment	1.49 (0.97–2.29)	.070	1.73 (1.10–2.70)	.017	1.60 (0.92–2.77)	.097
EBBS insertion disgust	0.91 (0.57–1.46)	.698	0.66 (0.42–1.04)	.070	0.41 (0.22–0.76)	.004
EBBS faecal disgust	1.47 (0.88–2.45)	.142	0.75 (0.46–1.24)	.265	1.12 (0.58–2.17)	.734

^1^ 0 = female (*n* = 233), 1 = male (*n* = 72); ^2^ 0 = not perceived low CRC risk (*n* = 236), 1 = perceived low CRC risk low risk (*n* = 69); ^3^ 0 = not perceived high CRC risk (*n* = 266), 1 = perceived high CRC risk (*n* = 39); ^4^ 0 = never discussed CRC risk with doctor (*n* = 207), 1 = had discussed CRC risk with doctor (*n* = 98)

Next, the predictors of reporting having previously provided a faecal sample were assessed. This model was significant at Step 1 χ^2^ (6, *N* = 304) = 34.35, *p* = .000 and explained 14.3% (Nagelkerke *R*^*2*^)of the variance in having ever provided a faecal sample; older age (Wald = 12.01, *p* = .001) and physician discussion (Wald = 8.08, *p* = .004) predicted a greater likelihood of having given a sample whereas, perceptions of low colorectal cancer risk were associated with a lower likelihood (Wald = 4.44, *p* = .035). The model was strengthened at Step 2 with the entry of the EBBS subscales, χ^2^ = 44.97, *p* = .000 and explained 18.5% of the variance (Nagelkerke *R*^*2*^). Having discussed risk with a doctor (Wald = 6.85, *p* = .009), older age (Wald = 9.35, *p* = .002), and greater embarrassment (Wald = 0.49, *p* = .017) predicted a greater likelihood of having provided a sample, whereas perceptions of lower risk (Wald = 4.29, *p* = .038) predicted, and greater insertion disgust (Wald = 3.28, *p* = .070) marginally predicted, a lower likelihood.

Finally, the predictors of reporting having had an invasive bowel investigation in the past 5 years were assessed. This model was significant at Step 1, χ^2^ (6, *N* = 304) = 95.32, *p* = .000 and explained 38.2% (Nagelkerke *R*^*2*^) of the variance; older age (Wald = 13.66, p =.000), having discussed screening with a doctor (Wald = 38.27, *p* = .000), and greater CRC knowledge (Wald = 4.15, *p* = .042), predicted a greater likelihood of reporting a prior investigation. The model was strengthened at Step 2, with the EBBS subscales χ^2^ = 107.22, *p* = .000 and explained 42.2% of the variance. Greater insertion disgust predicted a lower likelihood of reporting an invasive bowel screening test in the past 5 years (Wald = 8.09, *p* = .004). Older age (Wald = 9.90, *p* = .002) and having discussed risk with a doctor continued to predict greater likelihood (Wald = 33.96, *p* = .000), while CRC knowledge was no longer significant at this second step.

## Discussion

Participation rates of CRC screening programmes fall consistently below recommended levels and people delay presenting to medical care with bowel symptoms [34]; results of this research suggest that alongside demographic and medical variables, emotions may also be a factor. Unsurprisingly, age, discussions with a doctor, and perceptions of bowel cancer risk predicted reports of prior screening and decisions about help-seeking in a hypothetical scenario. More to the point, the addition of emotions consistently increased the  models’ ability to predict delay or bowel screening avoidance. Greater fear of a detection of cancer predicted the decision to delay attending a doctor’s clinic when confronted with suspicious bowel symptoms and greater insertion disgust predicted a lower likelihood of having had a prior bowel screening investigation. Interestingly, greater embarrassment was associated with a greater likelihood of reporting having provided a faecal sample in the past. Below, these findings are linked to prior work and discussed more fully, before the implications of these results for CRC screening programmes are considered.

Our findings extend previous work that identified the role of faecal disgust as a barrier to bowel screening [31] by introducing the possibility that specific emotional responses might impact screening behaviours in different ways, and that the source of emotion is important. Our data imply that fear, embarrassment, and disgust all have a role to play in our understanding of CRC screening avoidance. In each analysis, emotional barriers regarding testing or screening predicted screening over and above medical and demographic factors. For example, fear of a negative outcome was associated with greater avoidance where people imagined taking a container of faeces to a doctor’s clinic. Why it was this emotion rather than disgust in this scenario is unclear. Arguably, it might have been that the fear of a cancer diagnosis outweighed the potential disgust related to the collection of the faeces itself.

In contrast, when predicting prior CRC investigations, it was disgust related to anal insertions rather than to faecal exposure that best predicted avoidance. In some ways this finding is inconsistent with suggestions that faecal exposure is the central emotional impediment to bowel screening [35, 36], although prior studies have not separated these two sources of disgust response. Hence, whilst we do not discount the relevance of faecal aversion (particularly to some forms of testing), it might be that measures of faecal aversion co-vary with insertion concerns, or that for some tests concerns about faeces “pale in comparison” to feelings regarding inserting medical apparatus into the anus. Depending on the particular CRC screen that is being targeted, campaigns that seek to normalise exposure to faeces might be boosted with messages aimed at minimising concerns about anal insertions.

Interestingly, the direction of relationships differed across emotions and variables. As noted above, insertion disgust predicted a lower likelihood of having provided a faecal sample (i.e., greater avoidance), whereas embarrassment predicted a greater likelihood of having provided a sample (presumably, *less* avoidance). Whilst the latter finding seems initially counterintuitive, this result is not without precedent. In one study, while faecal/rectal embarrassment predicted a lower odds of a CRC screen, a more general embarrassment regarding examination intimacy predicted (albeit marginally) greater odds of a previous screen [15]. It might be that some forms of embarrassment are associated with greater negative affect or health anxiety, and it is this cluster of characteristics that makes a person more likely to screen (i.e., screening provides reassurance to those that worry). A further possibility is that the direction of the relationship between embarrassment and screening is the other way around—it is not that embarrassment predicts screening so much as previous exposure to faecal collection leads to a greater recognition of the embarrassment involved. Whilst evaluating these possibilities is beyond the scope of this work, our findings clearly reinforce the need to differentiate fear, disgust, and embarrassment in both measurement as well as their impact on cancer screening behaviours.

On the surface, identifying the ‘practical’ reasons for CRC screening non-attendance (access, transport etc.) should address impediments to screening. However, screening programmes have consistently struggled to recruit despite efforts to address these problems [7]. Data from this report suggest that emotions matter in CRC screening contexts. By understanding the specific emotions at play, messages and interventions can be tailored to address the most potent deterrents to screening behaviour. As noted, non-attendance due to fear of a diagnosis will require a different message than an intervention aimed at reducing avoidance due to disgust regarding the procedure itself [18]. Thus, interventions such as media campaigns, invitation letters, and communications from the staff who greet patients and carry out procedures could be tailored to address the particular emotional deterrents of fear, embarrassment, and disgust. Whilst, interventions that utilise such emotions have not been previously studied, recent research indicates that exposure to other emotions (i.e., anticipated regret) can increase CRC screening rates in people with low intentions to screen [17].

Although this study makes a useful contribution to the literature by demonstrating the role of fear, embarrassment and disgust in CRC investigations, it is not without its limitations. First, the timeline of data collection creates the possibility of historical confounds influencing responses. To check for this, we ran alternative analyses excluding the 48 participants gathered in 2014, and results remained the same apart from the expected marginalisation due to loss of power on two of the measures. Over the research time period no bowel screening programmes were being offered (aside from a single pilot programme in one of the local health boards), and so, would have been relatively unknown at the time. We therefore did not ask about intentions to screen, however, future research would ideally include an intention-to-screen question. Due to the way participants were recruited we were unable to estimate a response rate which limits our ability to know the extent to which our results might be biased. We also did not collect data about education level and so have no way of knowing whether the sample represents the CRC screening population. What we do know is that our gender split (76% female / 24% male) is not representative and, given the differences between men and women in response to cancer screening [37], this is a limitation of the work. The majority of our sample chose to complete the questionnaire online meaning we may have differentially accessed a more electronically-minded sample that does not necessarily reflect the population of people aged 45+. The study also employed a cross-sectional, retrospective design, and whilst emotions in this study predicted previous screening behaviours and responses to a hypothetical scenario, we cannot be sure whether precisely the same pattern would be found for future screens; there is certainly evidence for the inaccuracies in people’s reporting of their past screening uptake [38]. Future research would benefit by *prospectively* evaluating dispositional and attitudinal factors prior to patients being offered CRC screening and might also more systematically consider the difference between *anticipated* versus actual emotion. Bowel screening procedures can be anticipated to be much worse than the actual experience [39], and enquiry into the extent that such over-estimations in anticipated emotion influence future behaviours may be important.

Future research might also investigate the importance of ethnicity or race in analyses. Disparities in screening are well known [37, 40] and the populations with the poorest survival statistics typically have lower screening rates. The extent to which avoidance-promoting emotions play a greater or lesser role in these populations is an important question for future studies. It might also be that the language to describe these emotions might vary across populations. For instance, words like ‘nasty’ and ‘gross’ may not be the language of disgust across all groups. Research that identifies how communications and screening messages might be targeted to address emotional sensitivities has potential to increase screening among these at-risk groups.

## Conclusions

This report suggests that continuing to base CRC screening communications on the assumption that people behave rationally risks ignoring the elephant in the room—people get frightened, embarrassed, and disgusted by screenings and cancer. By understanding the emotional substrates for avoidance behaviours in CRC screening, we can tap into another layer of motivation to inform screening development, communication, and outreach. Importantly, however, it is not simply that emotions per se matter but, rather, that particular emotions matter—namely fear, embarrassment, and disgust. Understanding how specific emotions may impact screening decisions and behaviour is an important direction for future work.

## Abbreviations

CRC: **C**olorectal cancer
CT: **C**omputed tomography
DES: Differential emotions scale
DPSS: Disgust propensity and sensitivity scale – revised
DS-R: Disgust sensitivity – revised scale
EBBS: Emotional barriers to bowel screening scale
URL: Universal resource locator
